# Clinical Efficacy of Telemedicine for Musculoskeletal Conditions in a Medicare Advantage Population

**DOI:** 10.1089/tmr.2025.0017

**Published:** 2025-05-19

**Authors:** Mary I. O’Connor, Megan Dorak Ribaudo, Kaitlyn Cooney Peters, Jim Fiechtl, Tessy Oommen, Carrie McCulloch, Rusti Quarles, Krista Schonrock, Ryan A. Grant

**Affiliations:** ^1^ Vori Health, Nashville, Tennessee, USA.; ^2^ Select Health, Murray, Utah, USA.

**Keywords:** musculoskeletal, telemedicine, Medicare Advantage, integrated practice unit, multidisciplinary

## Abstract

**Introduction:**

Musculoskeletal (MSK) conditions are highly prevalent among the Medicare population, and telerehabilitation has been shown to be equivalent to traditional in-person physical therapy for many patients.

**Method:**

We studied the clinical outcomes of 100 consecutive Medicare Advantage patients from one insurer treated with a virtual physician-led MSK care team model. Patients had to have completed at least three clinical video visits to be eligible for participation. We also recorded rates of image ordering and referrals for in-person services.

**Results:**

The average age of our patients was 72 years with sex equally divided. Fifty-two percent of our patients presented with symptoms in their lower back. All patients had an initial evaluation with a physician or nurse practitioner (NP) and a physical therapist during the same video visit encounter. The average number of follow-up physical therapy visits was 7.6, and 14% had a follow-up visit with the physician or NP. Forty-six percent of patients saw a health coach and 15% a registered dietitian for concomitant concerns. Imaging studies were ordered in two patients and referrals for in-person services were made in three. With all care provided through a telemedicine platform, we found a high degree of pain improvement (83%, *p* < 0.001) and physical health improvement (84–86%) in our cohort.

**Conclusion:**

Our experience with Medicare Advantage patients demonstrates that multidisciplinary physician-led care in the telemedicine setting is effective in improving pain and physical function in older patients with MSK conditions.

## Introduction

The burden of musculoskeletal (MSK) pain is an underappreciated national health crisis for adults aged 65 and older (seniors) in the United States. The U.S. Centers for Disease Control and Prevention National Center for Health Statistics states that 45.6% of seniors will have experienced back pain, 50.3% lower limb pain, and 42.0% upper limb pain in the preceding 3 months.^1^ These prevalences are higher for women and non-Hispanic White patients, followed by non-Hispanic Blacks, Hispanics, and Asians.^1^ Moreover, many patients with MSK symptoms will also suffer from anxiety and depression. With total Medicare spending increased by 8.1% in 2023 and expected to continue to climb, advancing cost-effective methods to deliver high-quality MSK care to seniors is imperative.^2^

Telemedicine has emerged as a care delivery method to lower health care costs and provide convenience to patients.^3^ Telemedicine for MSK conditions can range from programs focused on a home-based exercise program and digital physical therapy, to those that offer diagnostic services as provided by a physician. Diagnostic concordance between telemedicine and in-person care for MSK conditions has been shown in a systematic review and meta-analysis to be “good to very good” with treatment plan concordance deemed to be “probably good to excellent.”^4^ In addition, physical therapist-led, exercise-based telerehabilitation had been shown to be noninferior to face-to-face physical therapy rehabilitation for many patients with MSK conditions.^5,6^

For seniors who bear a higher burden of MSK disease, the use of telemedicine by seniors is now common. In 2024, 47% of seniors reported being comfortable or very comfortable with video health care visits.^7^ In a systematic review of only randomized controlled trials in adults 60 years of age and older with MSK conditions, real-time telerehabilitation was equal in effectiveness to conventional face-to-face treatment in improving physical performance.^8^ Furthermore, patients 65 years of age and older are just as likely, if not more, to complete a digital MSK program (83% completion rate) compared to younger generations (67–82% completion rate).^9^ Rates of adherence, engagement, and satisfaction in a different digital MSK care program were also shown to be higher for adults 65 years of age or older compared to younger individuals.^10^

The value of multidisciplinary care for MSK patients is well documented.^11–15^ Research on multidisciplinary care for MSK patients in the telemedicine setting is limited. We previously published the positive results of our telemedical interdisciplinary care team approach in the management of adults with low back pain.^16^ In this study of a new cohort of patients, we sought to expand the literature on the clinical efficacy of telemedicine for MSK conditions. Given the prevalence of MSK disease in the senior population, we focused on a senior population, specifically a Medicare Advantage population, using our virtual physician integrated practice unit, which consists of an MSK trained physician, nurse practitioner (NP), physical therapist, health coach, and registered dietitian.

The primary aim of our study was to analyze our initial experience with Medicare Advantage patients in our telemedicine model. We wanted to gain insights into patient-reported outcomes for pain, anxiety, and depression, as well as patient self-reported assessment of their normal function for seniors with MSK conditions. Secondary aims included an assessment of the ordering of images by our clinicians as well as referral of patients for in-person medical services.

## Materials and Methods

A retrospective clinical review was conducted of the first 100 consecutive patients who met the inclusion criteria. Inclusion criteria were: (1) Medicare Advantage insurance with a single insurer, (2) baseline pain rating score ≥2, and (3) at least three completed virtual visits with Vori clinicians. Our study was reviewed by (The Institute for Evaluation and Research, LLC) TIER (Institutional Review Board) IRB and approved as exempt. Patients were excluded who did not meet these inclusion criteria.

As our goal was to study our initial experience with Medicare Advantage patients, we chose the first 100 consecutive patients as a potential method to reduce bias in patient selection. All patients were enrolled in a Medicare Advantage program offered by Select Health.

In the care model studied, each patient has an initial virtual evaluation performed by a specialty MSK physician (typically, Physical Medicine and Rehabilitation), or for a smaller number of patients by an experienced MSK NP, and a physical therapist who had a minimum of 5 years of MSK experience. Each initial visit was 40–45 min in duration and performed via one video link on our platform. During this single video visit, the physician (or NP) conducted an evaluation to render a medical diagnosis, and the physical therapist conducted an evaluation to render a functional diagnosis. This combined evaluation and assessment permitted the physician/NP and the physical therapist to create a personalized, evidence-based, biopsychosocial treatment plan during the same encounter.

During the initial visit, a medical history was obtained including screening for red flags, a physical examination was performed, a motivational interview was conducted to better understand the patient’s primary goals and assess yellow flags (psychological factors such as fears and unhelpful beliefs that may increase the risk of a negative outcome), pain education was provided, and instructions given on use of the platform’s AI computer vision motion-tracking program without wearable sensors to support their home exercise program. During their first visit, patients are also assessed in regard to meeting criteria for medical imaging and nonopioid prescriptions. Subsequent follow-up video visits were scheduled with physical therapy or the physician/NP as clinically appropriate. Virtual services with a health coach and/or registered dietitian were also provided, particularly for patients with modifiable health behaviors or conditions that likely negatively impacted their MSK condition (e.g., obesity, poor sleep, stress). Anchored by evidence-based guidelines, health coaching, and dietitian services were personalized to each patient based on their individual needs.

Medical questionnaires were sent to patients to be completed at the time of the initial evaluation, at 1 month of follow-up, 2 months of follow-up, 12 months of follow-up, or at the time of clinical discharge. Medical questionnaires included the numeric pain rating score in which patients were asked to rate their pain on a scale of 0 (no pain) to 10 (maximum pain). Generalized Anxiety Disorder 2-item (GAD-2) was used as a screening tool for anxiety with scores ranging from 0 to 6 with a score of 3 or higher having a sensitivity of 86% and specificity of 83% for the diagnosis of GAD.^17^ Patient Health Questionnaire 2 (PHQ-2) was used as a screening tool for depression with a score ranging from 0 to 6 with a score of 3 or higher likely indicating a major depressive disorder.^18^ PROMIS-10 was utilized and analyzed based on mental health and physical health subscores.^19^ The Single Assessment Numeric Evaluation (SANE) score was assessed at every physical therapy video visit. The SANE score represents the patient’s assessment of their joint function and pain on a scale of 0–100, with 100 representing normal function.^20^ Statistics were performed using IBM SPSS version 30 with before and after variables evaluated using a paired *t-*test with two-sided *p*-values. The standard deviation is abbreviated SD.

## Results

Our patient cohort consisted of the first 100 patients who had a first clinical visit starting July 27, 2023, and had a minimum of two additional clinical visits with a single insurer. Sex was equally divided between males and females. The average age of patients was 72 years (median 71, range of 59–86). The average BMI was 29.8 (range: 18.8–51.7), consistent with the high prevalence of MSK conditions in overweight populations.

**FIG. 1.**
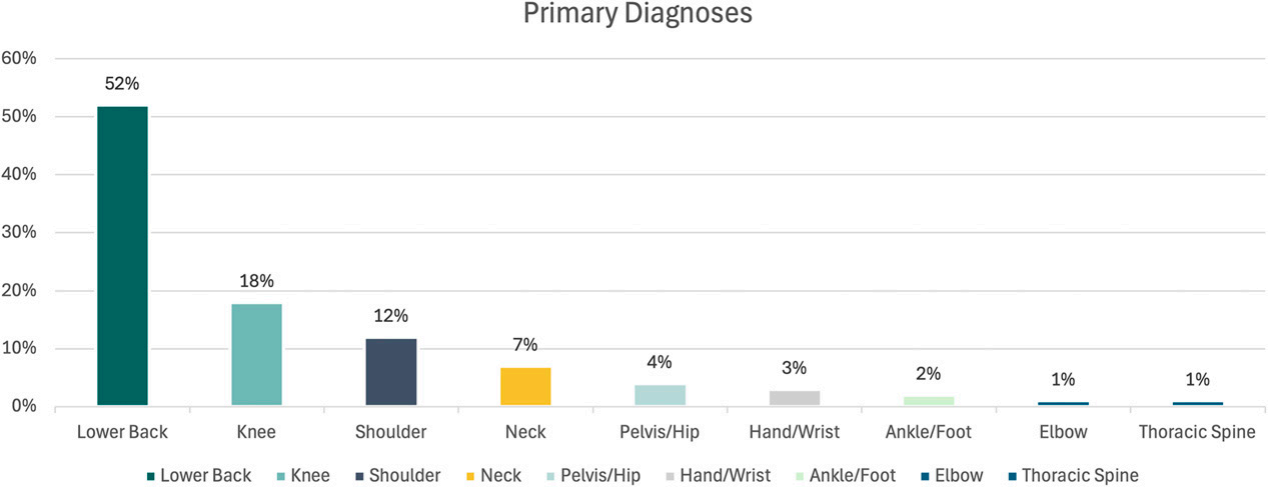
The body region of the primary diagnoses of the patients.

The primary ICD-10 diagnosis codes are listed in Table 1, and the corresponding body regions of the primary diagnoses are illustrated in (Fig. 1). A secondary MSK diagnosis was made in 38% of patients, distributed across these body regions.

**Table 1.** Primary ICD-10 Diagnostic Codes, Descriptions, and Anatomical Region Category for 100 Subjects

ICD-10 code	Code description	Category	#Patients
M54.50	Low back pain unspecified	Lower back	37
M25.519	Pain in an unspecified shoulder	Shoulder	6
M25.569	Pain in an unspecified knee	Knee	6
M47.816	Spondylosis w/o myelopathy or radiculopathy	Lower back	5
M54.2	Cervicalgia	Neck	5
M25.561	Pain in the right knee	Knee	4
M17.0	Osteoarthritis of knee	Knee	3
M25.511	Pain in the right shoulder	Shoulder	3
M25.562	Pain in the left knee	Knee	3
M51.36	Other intervertebral disc degeneration, lumbar region	Lower back	3
M54.16	Radiculopathy in the lumbar region	Lower back	3
M47.812	Spondylosis without myelopathy or radiculopathy, cervical region	Neck	2
M54.17	Radiculopathy in lumbosacral region	Lower back	2
M75.41	Impingement syndrome of right shoulder	Shoulder	2
M17.12	Unilateral primary osteoarthritis left knee	Knee	1
M17.31	Unilateral post-traumatic osteoarthritis, right knee	Knee	1
M19.041	Primary osteoarthritis, right hand	Hand/wrist	1
M25.512	Pain in the left shoulder	Shoulder	1
M25.529	Pain in the unspecified elbow	Elbow	1
M25.541	Pain in joints of the right hand	Hand/wrist	1
M25.552	Pain in the left hip	Pelvis/hip	1
M25.572	Pain in the left ankle and joints of the left foot	Ankle/foot	1
M25.631	Stiffness in the right wrist that is not classified elsewhere	Hand/wrist	1
M48.061	Spinal stenosis, lumbar region without neurogenic claudication	Lower back	1
M53.2X8	Spinal instabilities in the sacral and sacrococcygeal region	Pelvis/hip	1
M54.51	Vertebrogenic low back pain	Lower back	1
M54.6	Pain in thoracic spine	Thoracic spine	1
M67.951	Unspecified disorder of synovium and tendon, right thigh	Pelvis/hip	1
M76.02	Gluteal tendinitis left hip	Pelvis/hip	1
M79.671	Pain in the right foot	Ankle/foot	1

Medical questionnaire scores were obtained at baseline in nearly all patients, and final surveys were completed at various time intervals for a given outcome.

### Number of visits

**FIG. 2.**
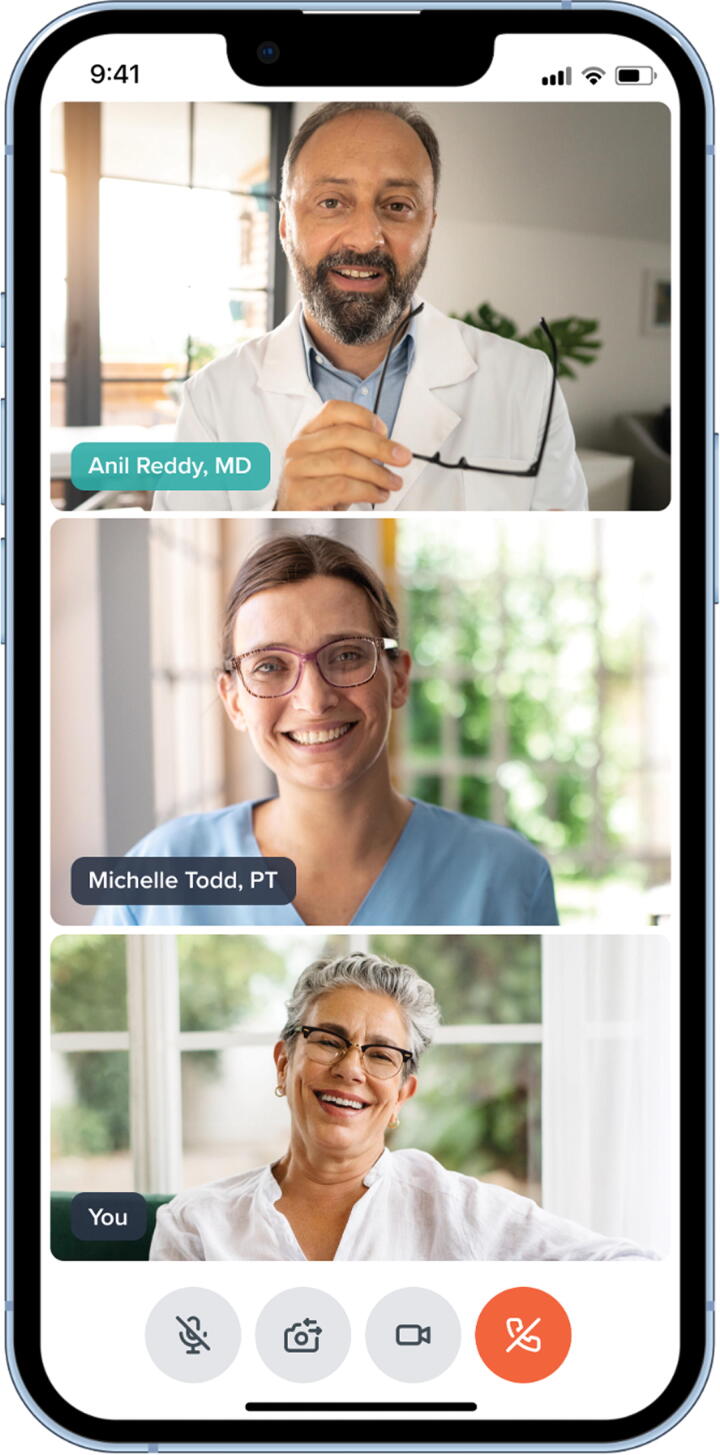
Representative image of the telemedicine evaluation of a patient with our physician and physical therapist.

After the initial evaluation visit by both the physician/NP and physical therapist (Fig. 2), patients had at least two additional video visits with clinicians. Follow-up physical therapy visits occurred in 99% of patients and averaged 7.6 visits (range: 1–30; median, 6). Fourteen percent had one follow-up virtual visit with the physician or NP, whereas 100% of patients had access to the physician or NP via chat. Nearly half of the patients had health coaching services, 47%, with an average number of visits of 7.6 (range: 1–25; median, 5). Finally, 15% of patients had services by a registered dietitian with an average number of visits of 5.3 (range: 1–9; median, 4).

**FIG. 3.**
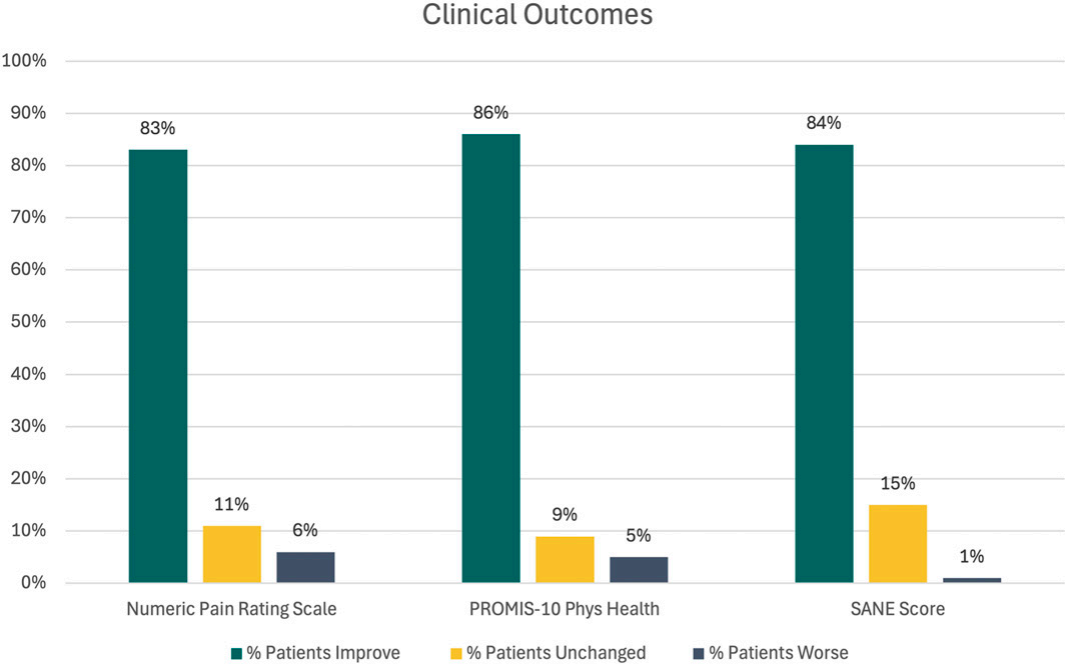
Significant improvement in pain and functional clinical outcomes.

### Pain improvement

All patients had baseline and follow-up pain scores at an average of 115 ± SD 93 days (range: 8–362 days). Pain improved in 83% of patients from a baseline average score of 4.6 ± 1.61 (range: 2–8) to the final score of 1.3 ± 1.47 (range: 0–7; *p* < 0.001; Fig. 3). Pain was unchanged in 11% of patients who had a baseline and final average pain score of 3.1 ± 1.14 (range: 2–6). The pain increased in six patients from a baseline average of 4.2 ± 1.47 (range: 2–6) to 6.0 ± 1.67 (range: 4–8; *p* = 0.012).

**FIG. 4.**
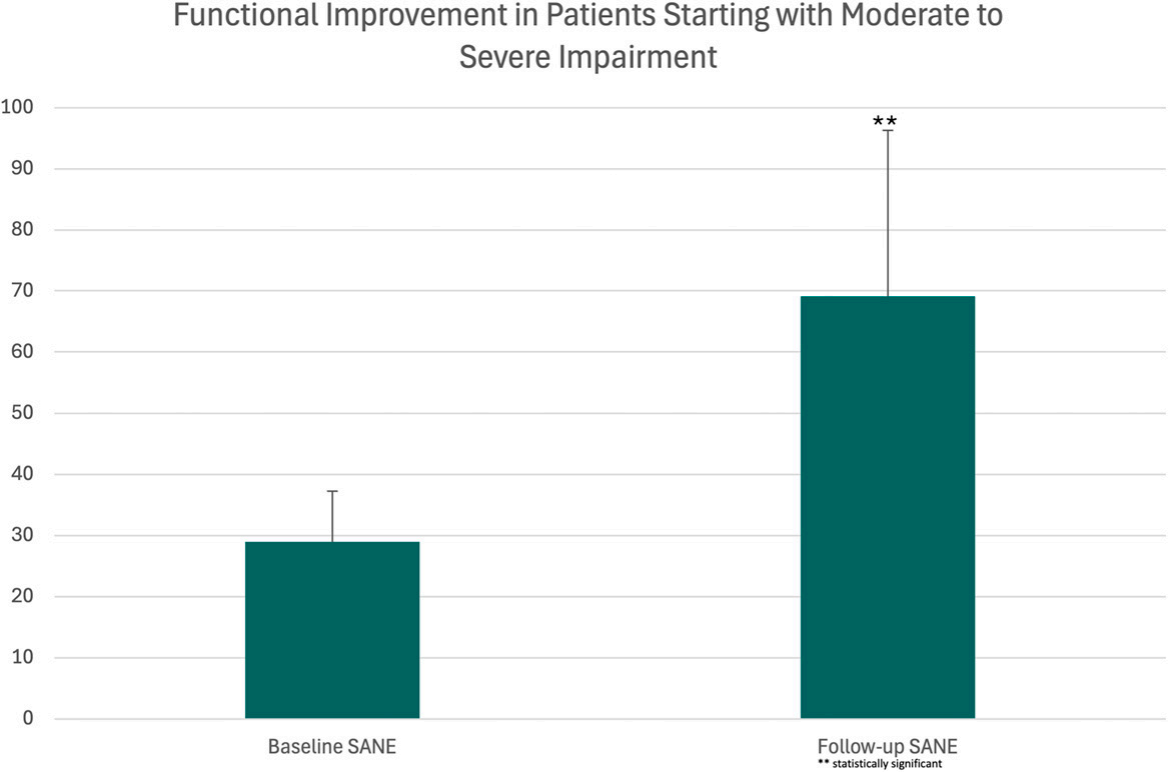
Improvement in SANE scores for patients with moderate to severe impairment at baseline, defined as a score of 40 or less. SANE, Single Assessment Numeric Evaluation.

### Physical health

Baseline PROMIS-10 Physical Health T-scores were available in 96 patients with final scores in 57 patients with an average follow-up of 87 days ± 58.5 (range: 15–308 days). The baseline T-score average was 42.7 ± 7.7, consistent with mild physical impairment, and the average follow-up score was 48.2 ± 7.3 (*p* < 0.001). At follow-up 49 of the 57 (86%) patients had improved their T-score by an average of six points, moving them into the normal range (Fig. 3). In five patients there was no change in T-score and in three the T-score worsened from 42.4 ± 13.5 to 39.5 ± 12.9 (*p* = 0.023).

SANE scores were available in 82 patients at baseline and at final follow-up at an average of 112 days ± 105.8 (range: 7–394 days). Scores improved in 84% of patients from an average baseline of 56.1 ± 20.6 to 79.4 ± 19.4 (*p* < 0.001; Fig. 3). Only one patient showed a decrease in score from 85 to 80, which is not significant, as scores above 80 represent near-normal function. For patients with moderate to severe impairment, defined as a SANE score of 40 or less, 29% of patients scored below 40 with an average score of 28.9 ± 8.1 (range: 10–40). At follow-up these SANE scores improved significantly to a final average SANE score of 69.1 ± 27.2 (*p* < 0.001; Fig. 4). To try and understand if patients who had shorter courses of treatment or shorter follow-up had clinical improvement, we also analyzed patients with follow-up less than 21 days (12 patients). This cohort had an average baseline score of 63.3 ± 21.98 (range: 20–90) and significantly improved to an average of 76.6 ± 27.8 (*p* = 0.022). Of this cohort, no patient scored worse than their baseline score.

### Mental health

As depression and anxiety are common comorbidities in patients with MSK conditions, we screen for anxiety and depression at baseline with GAD-2, PHQ-2, and PROMIS-10 Mental Health questionnaires. All 100 patients completed baseline GAD-2 (anxiety screening tool) and PHQ-2 (depression screening tool), both have a maximum score of 6. Follow-up GAD-2 and PHQ-2 scores were available in 70 patients at an average of 109.1 ± 105.3 days (range: 10–435 days).

The average baseline GAD-2 score was 0.61 ± SD 0.91 (range: 0–5) and on follow-up 0.47 ± SD 0.77 (range: 1–3) with p = 0.14. Given that GAD-2 has a sensitivity and specificity for diagnosing anxiety starting at score 3 and higher, there was no significant anxiety at baseline or in follow-up. No patients had a statistically significant increase in GAD-2 scores during treatment. There were no final scores higher than 3.

The baseline PHQ-2 score averaged 0.66 ± SD 0.88 (range: 0–5) and on follow-up 0.40 ± 0.73 (range: 0–3) with *p* = 0.021. Given that PHQ-2 has sensitivity and specificity for diagnosing depression starting at scores 3 and higher, there was overall no significant depression at baseline or in follow-up. In the 70 patients with follow-up surveys, all patients who had scores of 3 or higher decreased below 2, which is congruent with the statistically significant *p*-value results from the baseline and follow-up depression scores. Only one patient had an increase in score going from a 2 to a 3. There were no final scores higher than 3.

A total of 12 unique patients had a flagged score on GAD-2, PHQ-2, or both for a score of 3 or higher. Of these 12 patients, 5 patients consented to dedicated cognitive behavioral coaching services, and final scores were improved by 2 points in the 2 patients with follow-up scores. We contact each patient with a GAD-2 or PHQ-2 score of 3 or higher to assess for the potential for self-harm and recommend mental health services. We share these questionnaire results with the patients’ PCPs.

Finally, baseline PROMIS-10 Mental Health T-scores averaged 50.1 ± 8.4 (range: 12–68), and follow-up scores in 62 patients demonstrated improvement to an average T-score of 52.3 ± 7.1 (range: 28–68; *p* = 0.013). A score of 50 or higher is considered normal.

### Ordering of imaging and referral for in-person services

Our clinicians ordered only two imaging studies on these 100 patients, namely radiographs of the knee and lumbar spine. Three patients were referred for in-person care, including one each for total knee arthroplasty consultation, interventional pain management consultation, and concomitant (hybrid) in-person physical therapy.

## Discussion

This experience supports the clinical efficacy of our telemedicine model for individuals with MSK conditions and Medicare Advantage insurance. In our consecutive cohort of 100 MSK patients with an average age of 72 and a minimum of three video clinical visits, pain improved in 84% of patients and physical health in 84–86% based on SANE and PROMIS-10, respectively. There was no statistically significant worsening in any patient’s pain, and a small decrease in physical function occurred in less than 3% of patients. Given that 52% of patients in this study presented for evaluation and treatment of low back pain, consistent with the known prevalence of this condition in the senior population, we believe our cohort is generally representative of a typical Medicare Advantage population.^1^

The improvements in both pain and physical function seen in this study were achieved with a variable number of virtual visits with various clinicians. The core clinical services were provided by a physician or NP and a physical therapist during the initial evaluation.

Patients had an average of 7.6 follow-up virtual physical therapy visits, evidence that many seniors are comfortable with telerehabilitation and consistent with published studies,^5,10,21^ and slightly less than 9.1 average visits for a Medicare population of only low back pain patients.^22^ We believe the consistency and focus in our model on patient engagement with the physician/NP and physical therapist is responsible for the 84% improvement in pain and 84–86% improvement in physical function in our cohort.

Given the multiple comorbidities in the Medicare population, health coaching was heavily utilized by this patient cohort, in which 46% of patients engaged directly with a health coach as part of their physician-led team. A small percentage of patients (15%) also received care from our registered dietitian, typically related to a diagnosis of obesity and comorbid nutritional ailments affecting the patients’ MSK condition. Our GAD-2 and PHQ-2 scores demonstrate that this patient cohort had no significant baseline anxiety or depression to start, with average starting and follow-up scores below 1. All patients who initially had scores of 3 or higher decreased below 2, which is congruent with our statistically significant *p*-value result of improvement from baseline to follow-up depression scores. Only one patient had an increase in score going from a 2 to a 3.

Even with no significant depression or anxiety in this cohort, the PROMIS-10 Mental Health scores still demonstrated a small but statistical improvement in scores, which further showcases the power of an integrated care team to improve scores already in the normal range.

Our experience with this cohort shows low rates of our clinicians ordering imaging. Only two patients had plain radiographs ordered by our clinicians. We focus on practicing evidence-based medicine and in the absence of trauma with concern for fracture or “red flag” findings worrisome for serious pathology, imaging is not needed for the initial evaluation and treatment of MSK patients.^23^ Inappropriate imaging for MSK patients increases health care costs and can lead to unnecessary procedures.^24^ The findings in this study are consistent with our published experience with very low rates of imaging for low back pain patients.^16^

### Limitations

Our study has limitations. One key limitation is the lack of a comparator population of patients who received in-person care. Furthermore, we have no control population of patients who received care in an in-person integrated practice unit model. Thus, we cannot compare our results with in-person services.

Our results may not generalize to the population of Medicare Advantage patients. Our subjects were all enrolled in one specific Medicare Advantage program, and our study sample is small. Some other Medicare Advantage populations may differ in their incidence of MSK conditions as compared to our cohort. Some Medicare Advantage patients may have limited access to or comfort with the technology required for telemedicine visits.

Other limitations include variability in the clinical follow-up length of time in our study. We chose to include a consecutive cohort of patients who met the inclusion criteria and did not define a minimum follow-up time for inclusion in the study. Except for pain scores, we lacked follow-up survey scores on every patients. We were not systematic in recommending health coaching services to those with abnormal mental health scores, highlighting an opportunity for us.

Finally, the distribution of diagnoses in our patients was skewed toward lumbar spine pathology, potentially limiting the application of findings to other anatomical regions. Given the number of patients in our cohort (100 patients), statistical analysis of those with low back pain (52%) compared to all others (48%) would have limited meaning. Furthermore, the variance in diagnoses (Table 1), even within the patients with low back pain, limits meaningful comparison.

## Conclusions

Our early experience with Medicare Advantage patients shows that multidisciplinary physician-led care in the virtual setting is very effective in improving pain and physical function in patients with MSK conditions with a low rate of ordering imaging studies.

## Informed Consent Statement

A waiver of informed consent by the study participants was granted to the authors by TIER IRB.

## References

[1] Lucas JW, Connor EM, Bose J. Back, Lower Limb, and Upper Limb Pain Among U.S. Adults, 2019. Available from: https://www.cdc.gov/nchs/products/databriefs/db415.htm [Last accessed: March 29, 2025]. 34473621

[2] NHE Fact Sheet. Available from: https://www.cms.gov/data-research/statistics-trends-and-reports/national-health-expenditure-data/nhe-fact-sheet [Last accessed: March 29, 2025].

[3] Ezeamii VC, Okobi OE, Wambai-Sani H, et al. Revolutionizing healthcare: How telemedicine is improving patient outcomes and expanding access to care. Cureus 2024;16(7):e63881; doi: 10.7759/cureus.63881 39099901 PMC11298029

[4] Vincent R, Charron M, Lafrance S, et al. Investigating the use of telemedicine by health care providers to diagnose and manage patients with musculoskeletal disorders: Systematic review and meta-analysis. J Med Internet Res 2024;26:e52964; doi: 10.2196/52964 39312765 PMC11459102

[5] Wicks M, Dennett AM, Peiris CL. Physiotherapist-led, exercise-based telerehabilitation for older adults improves patient and health service outcomes: A systematic review and meta-analysis. Age Ageing 2023;52(11):afad207; doi: 10.1093/ageing/afad207 37979183 PMC10657214

[6] Molina-Garcia P, Mora-Traverso M, Prieto-Moreno R, et al. Effectiveness and cost-effectiveness of telerehabilitation for musculoskeletal disorders: A systematic review and meta-analysis. Ann Phys Rehabil Med 2024;67(1):101791; doi: 10.1016/j.rehab.2023.101791 38128150

[7] AARP. (2024). *Telehealth and the changing landscape of health care* (Publication No. RES-00789-001). https://www.aarp.org/content/dam/aarp/research/topics/health/coverage-access/telehealth-health-care.doi.10.26419-2fres.00789.001.pdf [Last accessed: March 29, 2025].

[8] Jirasakulsuk N, Saengpromma P, Khruakhorn S. Real-Time telerehabilitation in older adults with musculoskeletal conditions: Systematic review and meta-analysis. JMIR Rehabil Assist Technol 2022;9(3):e36028; doi: 10.2196/36028 36048520 PMC9478822

[9] Wang G, Bailey JF, Yang M, et al. Older adult use and outcomes in a digital Musculoskeletal (MSK) program, by generation. Front Digit Health 2021;3:3:693170; doi: 10.3389/fdgth.2021.693170 PMC8521841 34713170

[10] Areias AC, Janela D, Molinos M, et al. Managing musculoskeletal pain in older adults through a digital care solution: Secondary analysis of a prospective clinical study. JMIR Rehabil Assist Technol 2023;10:e49673; doi: 10.2196/49673 37465960 PMC10466151

[11] Jayakumar P, Moore MLG, Bozic KJ. Team approach: A multidisciplinary approach to the management of hip and knee osteoarthritis. JBJS Rev 2019;7(6):e10; doi: 10.2106/JBJS.RVW.18.00133 31246861

[12] Feng JE, Novikov D, Anoushiravani AA, et al. Team approach: Perioperative optimization for total joint arthroplasty. JBJS Rev 2018;6(10):e4; doi: 10.2106/JBJS.RVW.17.00147 30300250

[13] Morris JC, Moore A, Kahan J, et al. Integrated fragility Hip fracture program: A model for high quality care. J Hosp Med 2020;15(8):461–467; doi: 10.12788/jhm.3365 32118555

[14] Dlott CC, Moore A, Nelson C, et al. Preoperative risk factor optimization lowers hospital length of stay and postoperative emergency department visits in primary total Hip and Knee arthroplasty patients. J Arthroplasty 2020;35(6):1508–1515.e2; doi: 10.1016/j.arth.2020.01.083 32113812

[15] Davin S, Lapin B, Mijatovic D, et al. Comparative effectiveness of an interdisciplinary pain program for chronic low back pain, compared to physical therapy alone. Spine (Phila Pa 1976) 2019;44(24):1715–1722; doi: 10.1097/BRS.0000000000003161 31794508

[16] Woznica DN, Milligan M, Krymis H, et al. Telemedical interdisciplinary care team evaluation and treatment of people with low back pain: A retrospective observational study. Arch Rehabil Res Clin Transl 2023;5(3):100269; doi: 10.1016/j.arrct.2023.100269 37744196 PMC10517362

[17] Generalized Anxiety Disorder 2-item (GAD-2). Available from: https://www.hiv.uw.edu/page/mental-health-screening/gad-2 [Last accessed: March 29, 2025].

[18] Patient Health Questionnaire-2 (phq-2). Available from: https://www.hiv.uw.edu/page/mental-health-screening/phq-2 [Last accessed: March 29, 2025].

[19] Hays RD, Bjorner JB, Revicki DA, et al. Development of physical and mental health summary scores from the Patient-Reported Outcomes Measurement Information System (PROMIS) global items. Qual Life Res 2009;18(7):873–880; doi: 10.1007/s11136-009-9496-9 19543809 PMC2724630

[20] Owens BD. Staying SANE. Am J Sports Med 2021;49(14):3780–3782; doi: 10.1177/03635465211059123 34855546

[21] Summers SH, Gnecco T, Slotkin EM, et al. Significant cost savings and improved early clinical outcomes in medicare patients utilizing a clinician-controlled telerehabilitation system following total knee arthroplasty. J Arthroplasty 2024;39(8S1):S137–S142; doi: 10.1016/j.arth.2024.02.040 38401615

[22] The Moran Company. Available from: https://www.aptqi.com/Resources/documents/APTQI-Complete-Study-Physical-Therapy-Episodes-Lumbago-October-2017.pdf [Last accessed: March 29, 2025].

[23] Downie A, Hancock M, Jenkins H, et al. How common is imaging for low back pain in primary and emergency care? Systematic review and meta-analysis of over 4 million imaging requests across 21 years. Br J Sports Med 2020;54(11):642–651; doi: 10.1136/bjsports-2018-100087 30760458

[24] Karel YH, Verkerk K, Endenburg S, et al. Effect of routine diagnostic imaging for patients with musculoskeletal disorders: A meta-analysis. Eur J Intern Med 2015;26(8):585–595; doi: 10.1016/j.ejim.2015.06.018 26186812

